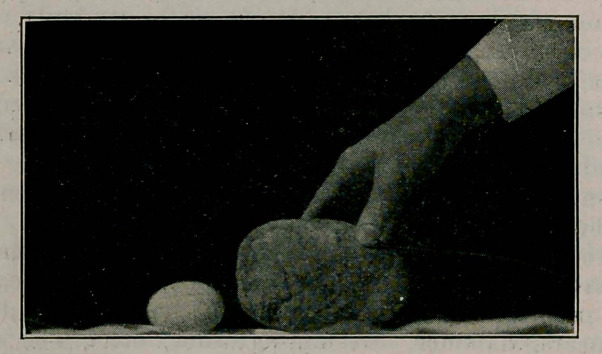# Vesical Calculi

**Published:** 1918-11

**Authors:** 


**Vesical Calculi.** C. E. Park of Prae, Siam, Int. Jour. of Surg., April, reports a native farmer, aged 24 who received a wound of the thigh 4 inches below the pubic bone, 12 years previously, in diving and striking a snag of teak wood. The wound discharged for a year; thereafter there was no complaint except of slight pain in the lower quadrant of the abdomen on exercise, till 3 years ago when dysuria and foul urine were noticed. The urethral sound clicked against a calculus which was operated upon. The calculus was encysted in a cancerous mass. The stone measured $2\frac{1}{2}$x$1\frac{3}{4}$ inch and weighed 4 drachms and had as a nucleus, a piece of teak $1\frac{1}{4}$ inch long. The patient recovered from the operation but died later of cancer.

D. W. MacKenzie of Montreal reports a calculus in a farmer aged 55, symptoms dating back 20 years, palpable bimanually, demonstrated by X-ray and successfully removed. It was ovoid, $17\frac{1}{2}$ to $12\frac{1}{2}$ inches in circumference, weight $38\frac{1}{2}$ ounces.

## Figures and Tables

**Figure f1:**
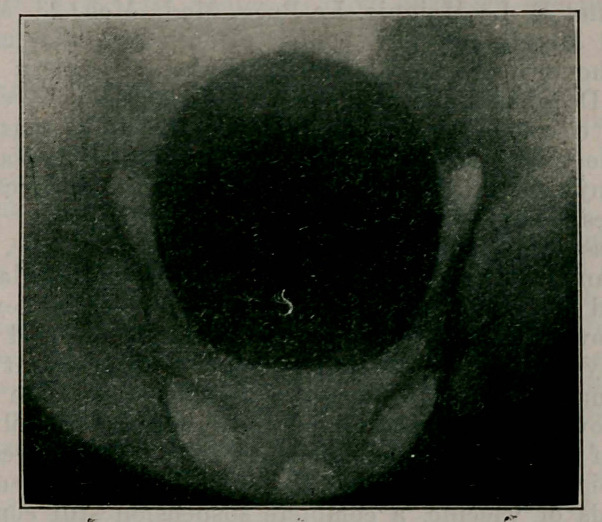


**Figure f2:**